# High red blood cell distribution width as a marker of hospital mortality after ICU discharge: a cohort study

**DOI:** 10.1186/s40560-018-0343-3

**Published:** 2018-11-16

**Authors:** Rafael Fernandez, Silvia Cano, Ignacio Catalan, Olga Rubio, Carles Subira, Jaume Masclans, Gina Rognoni, Lara Ventura, Caroline Macharete, Len Winfield, Josep Mª. Alcoverro

**Affiliations:** 1 Intensive Care Department, Hospital Sant Joan de Deu – Fundacio Althaia, Dr. Joan Soler 1, 08243 Manresa, Spain; 2 Universitat Internacional de Catalunya, Barcelona, Spain; 3 CIBERES, Madrid, Spain

**Keywords:** Scoring systems, Mortality prediction, Biomarkers

## Abstract

**Background:**

High red blood cell distribution width (RDW) is associated with worse outcome in diverse scenarios, including in critical illness. The Sabadell score (SS) predicts in-hospital survival after ICU discharge. We aimed to determine RDW’s association with survival after ICU discharge and whether RDW can improve the accuracy of the SS.

**Design:**

Retrospective cohort study. Setting: general ICU at a university hospital.

**Patients:**

We included all patients discharged to wards from January 2010 to October 2016.

**Methods:**

We analyzed associations between RDW and variables recorded on admission (age, comorbidities, severity score), during the ICU stay (treatments, complications, length of stay (LOS)), and at ICU discharge (SS). The primary outcome was hospital mortality. Statistical analysis included multivariable logistic regression and receiver operating characteristic curve (ROC) analyses.

**Results:**

We discharged 3366 patients to wards; median ward LOS was 7 [4–13] days; ward mortality was 5.2%. Mean RDW at ICU discharge was 15.4 ± 2.5%. Ward mortality was higher at each quartile of RDW (0.7%, 2.9%, 7.5%, 10.3%; area under ROC 0.81). A logistic regression model with Sabadell score obtained an excellent accuracy for ward mortality (area under ROC 0.863), and the addition of RDW slightly improved accuracy (AUROC 0.890, *p* < 0.05). Recursive partitioning demonstrated higher mortality in patients with high RDW at each SS level (1.6% vs. 0.3% in SS0, 9.7% vs. 1.1% in SS1, 21.9% vs. 9.7% in SS2), but not in SS3.

**Conclusion:**

High RDW is a marker of severity at ICU discharge and improves the accuracy of Sabadell score in predicting ward mortality except in the more extreme SS3.

## Introduction

The primary mission of intensive care units (ICU) is to improve the survival of critically ill patients. The first severity scores to predict mortality in patients admitted to the ICU were based on the maximum deviations of physiological variables. These scores have been periodically refined by including new variables or by adjusting the weight of each variable. In the most recent versions, the weight of physiological variables has decreased while the weight of patients’ comorbidities and health status has increased [1].

One physiological variable with the potential to improve the accuracy of severity and prognostic scores is red blood cell distribution width (RDW). Simple and inexpensive to obtain, RDW reflects the degree of heterogeneity of erythrocyte volume (anisocytosis); RDW has traditionally been used in the differential diagnosis of anemias. An increased RDW mirrors a profound deregulation of erythrocyte homeostasis involving both impaired erythropoiesis and abnormal red blood cell survival, which may be attributed to a variety of underlying metabolic abnormalities such as shortening of telomere length, oxidative stress, inflammation, poor nutritional status, dyslipidemia, hypertension, erythrocyte fragmentation, and alteration of erythropoietin function [2]. Many clinical conditions are associated with RDW above the upper limit, commonly accepted as 14.5%. Some of these conditions are inflammatory, others are hematological, and others are comorbidities (e.g., obesity, aging, smoking) [2]. High RDW has been associated with worse outcome in various conditions, such as community-acquired pneumonia [3, 4], myocardial infarction, cardiac arrest [5], and cerebral infarction [6], and in very different scenarios (e.g., critically ill patients on admission [7–9], during the ICU stay [10, 11], and long-term after hospital discharge [12]). Nevertheless, adding high RDW failed to improve the accuracy of common severity scores [13], suggesting that factors associated with high RDW (e.g., fever, leukocytosis, or cancer) may already be included in severity scores.

Our group investigates outcomes of patients in the ward after ICU discharge; this research led to the Sabadell score in 2006 [14]. A multicenter validation study confirmed that the Sabadell score classifies patients into four groups with very different ward mortality [15]. A trial is underway to elucidate whether intensivist surveillance can improve ward survival in different Sabadell score groups [16]. The real value of studying this population is that ICU surveillance after discharge is a growing wave, but needs to clearly frame those patients with a higher likelihood for complications and mortality. The follow-up of the entire ICU-discharged population is not worthy and unrealistic. Outreach teams need sensitive markers to identify ward patients at risk, and biomarkers like RDW are promising candidates [17, 18].

We aimed to determine the association of RDW with ward survival after ICU discharge and whether RDW can improve the accuracy of the Sabadell score. We hypothesized that adding RDW would improve the accuracy of the Sabadell score for predicting ward mortality after ICU discharge.

## Methods

We retrospectively studied all patients registered in our computerized database between January 2010 and October 2016; accordingly, informed consent was waived.

Our 16-bed mixed ICU admits patients with all kinds of medical and surgical conditions except those who have undergone cardiac surgery, neurosurgery, or transplantation and also serves as a coronary unit and stroke unit for a population of 200,000 inhabitants.

We excluded patients who died during the ICU stay and those who were not discharged to the ward, mainly due to transfer to other hospitals or nursing homes or direct discharge to their homes. Patients needing ICU readmission during the index hospitalization were not included twice; their outcomes were attributed to their first ICU admission.

On ICU admission, we recorded age, sex, diagnosis, SAPS3 score, and comorbidities. During the ICU stay, we recorded major ICU procedures (mechanical ventilation, renal replacement therapy, vasoactive drugs, tracheostomy, and blood transfusion) and adverse events (nosocomial pneumonia, acute renal failure, and delirium). At discharge from the ICU (or step-down unit, when applicable), the attending physician classified the patient according to the Sabadell score, a subjective tool explained in detail elsewhere [14]. Briefly, it has four levels of expected prognosis based on the clinician perception taking into account the illness evolution, comorbidities, sequelae, and socio-familiar support: score 0 is for patients with good prognosis, score 1 is for patients with poor prognosis in the medium-to-long term and acceptable ICU readmission, score 2 is for patients with poor prognosis in the short-term and debatable ICU readmission, and score 3 is for patients who are expected to die before discharge from the hospital.

Routine laboratory reports of blood work ordered by attending physicians as clinically required always included RDW. Based on previous references (4, 9, 10), high RDW was defined as greater than 14.5%. For the purpose of the present study, we used the last RDW recorded before discharge to the ward.

Variables are reported as means ± standard deviations, medians [interquartile ranges], or percentages and odds ratios, as appropriate. We used *t* tests to compare the means of continuous normal variables, Mann-Whitney *U* tests to compare the medians of continuous non-normal variables, and Fisher’s exact test to compare categorical variables; significance was set at *p* < 0.5.

Variables at ICU admission, during ICU stay, and at ICU discharge were compared between patients with and without high RDW values. Variables that were significant in the univariable analyses were included in a multivariable logistic regression analysis with ward mortality as the dependent (outcome) variable. The discrimination of the multivariate model was assessed using the area under the receiver operating characteristic curve (AUROC). Accuracy was considered to be good if the area under the curve was more than 0.75 and excellent if more than 0.85. To evaluate the contribution of high RDW to the prognostic value, we calculated the increase in the AUROC of the logistic model after including high RDW. Areas under the ROC curves (AUC) from logistic models were compared with Stata’s “roccomp” command (chi-squared test).

## Results

In the 6-year study period, we admitted 4675 patients to our ICU. Of these, 621 (13.3%) died during the ICU stay, 349 (7.5%) were directly discharged home, 329 (7.0%) were transferred to other hospitals or nursing homes, 10 (0.2%) were lost, and 3366 (72.0%) were discharged to the ward. The median length of ward stay was 7 [4–13] days, and 177 (5.3%) patients died in the wards.

Table 1 compares the recorded variables in patients with high RDW versus patients without high RDW. All the variables recorded at ICU admission (age, cancer, hepatic cirrhosis, and SAPS3 mortality risk), during ICU stay (blood transfusion, delirium, vasoactive drugs, mechanical ventilation, and acute renal failure), and at ICU discharge (ICU length of stay and Sabadell score) were worse in patients with high RDW, suggesting a profile of sicker patients.

**Table 1:** Characteristics on admission, during the intensive care unit (ICU) stay, at ICU discharge, and at hospital discharge in patients with normal versus high red cell distribution width

Variable	Normal RDW (≤ 14.5%) *n* = 1525	High RDW (> 14.5%) *n* = 1841	*p*
At ICU admission			
Age	61.8 ± 17.5	67.7 ± 15.1	< 0.001
Female sex	502 (33%)	742 (40%)	< 0.001
Cancer	86 (5.6%)	298 (16.2%)	< 0.001
Hepatic cirrhosis	14 (0.9%)	106 (5.8%)	< 0.001
SAPS3 mortality risk, %	11 [5.0–21.9]	25 [12.6–44.6]	< 0.001
During ICU stay			
Blood transfusion	45 (2.9%)	347 (18.8%)	< 0.001
Delirium	68 (4.5%)	144 (7.8%)	< 0.001
Vasoactive drugs	209 (14%)	608 (33%)	< 0.001
Mechanical ventilation	239 (16%)	585 (32%)	< 0.001
Acute renal failure	170 (11%)	533 (29%)	< 0.001
At ICU discharge			
ICU length of stay, days	2.3 [1.6–4.0]	3.3 [1.9–5.9]	< 0.001
Sabadell score:			< 0.001
0	1272 (79.8%)	1084 (57.4%)	
1	176 (11.0%)	465 (24.6%)	
2	62 (3.9%)	216 (11.4%)	
3	15 (0.9%)	76 (4.0%)	
Red cell distribution width, %	13.7 ± 0.54	16.9 ± 2.56	< 0.001
At hospital discharge:			
Ward length of stay, d	6 [4–10.5]	9 [5–16]	< 0.001
Ward mortality	21 (1.3%)	155 (8.2%)	< 0.001

*RDW* red cell distribution width, *SAPS3* Simplified Acute Physiologic Score 3, *ICU* intensive care unit

**Fig. 1:**
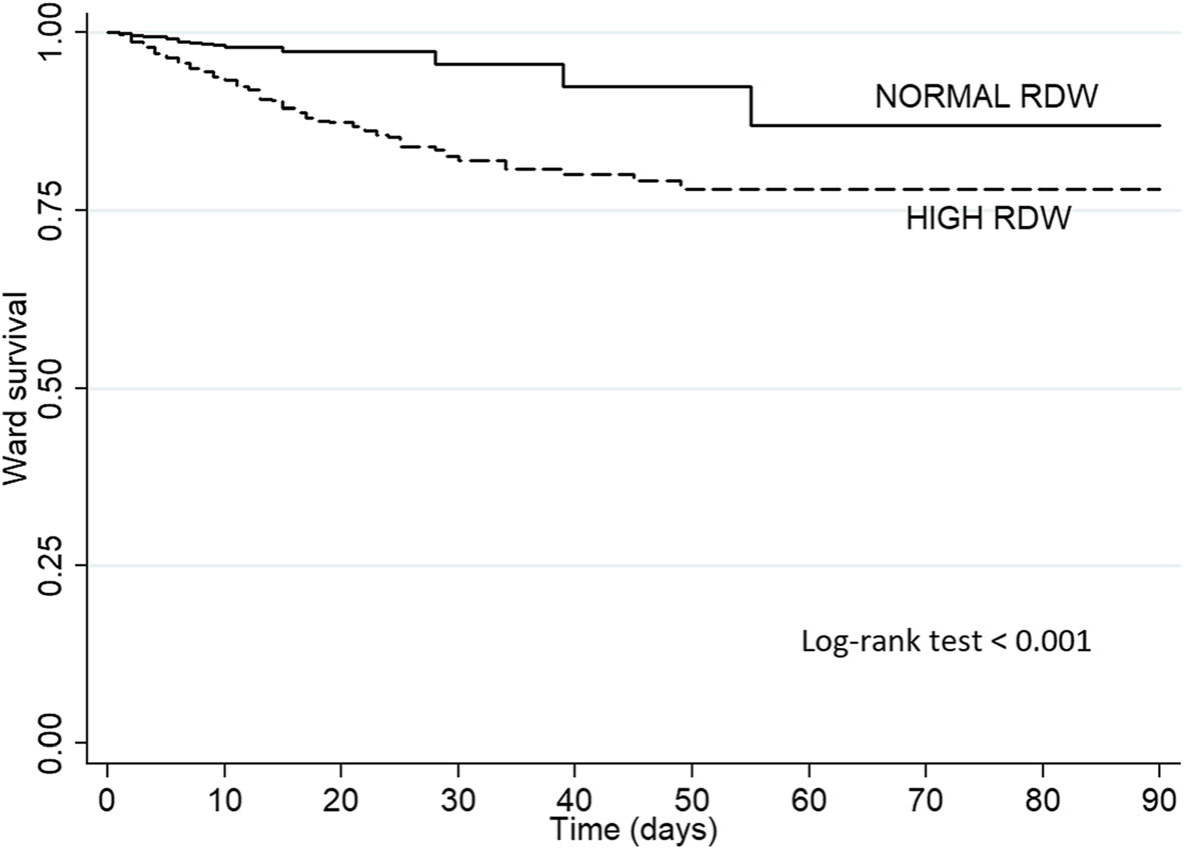
Kaplan-Meier curve of ward mortality of patients grouped as normal (dashed line) and high red blood distribution width (solid line)

After ICU discharge, patients with high RDW had longer ward stay (9 [5–16] vs. 6 [4–10.5] days; *p* < 0.001) and higher ward mortality (154 (8.4%) vs. 22 (1.4%); *p* < 0.001), as depicted in Fig. 1. For ward mortality, high RDW showed a sensitivity of 0.87 (0.81–0.92), specificity of 0.471 (0.468–0.474), positive predictive value of 0.084 (0.078–0.088), and negative predictive value 0.986 (0.979–0.991). A closer look at RDW and ward mortality demonstrated a stepwise correlation between RDW and ward mortality (Table 2). Partitioning the total population in quartiles of RDW, ward mortality ranged from 0.7% for RDW < 13.8% to 10.3% for RDW > 16.1%. The SAPS3 score was progressively worse at each quartile, and the standardized mortality ratio was also worse at higher quartiles of RDW (Table 2).

**Table 2:** Ward mortality, severity score on admission, and standardized mortality ratio for patients classified by quartiles of red cell distribution width at ICU discharge

Quartile	Red cell distribution width	Ward mortality	SAPS3 predicted risk of death	Standardized mortality ratio
1	< 13.8%	6 (0.7%)	14%	0.13
2	13.9–14.8%	25 (2.9%)	21%	0.32
3	14.9–16.3%	59 (7.5%)	28%	0.50
4	> 16.1%	86 (10.3%)	34%	0.77

*SAPS3* Simplified Acute Physiologic Score 3, *ICU* intensive care unit

Variables associated with ward mortality in the univariable analysis were age, cancer, SAPS3, delirium, acute renal failure, blood transfusion, vasoactive drugs, mechanical ventilation, ICU length of stay, Sabadell score, and high RDW (Table 3).

**Table 3:** Variables associated with ward mortality

Variable	Univariable OR	AUC	*p*	Multivariable OR	*p*
Age, years	1.07 [1.06–1.09]	0.73	< 0.001	1.03 [1.02–1.05]	< 0.001
Cancer	2.1 [1.4–3.1]	0.55	< 0.001		
SAPS3	1.04 [1.03–1.05]	0.78	< 0.001	1.012 [1.004–1.021]	< 0.005
Delirium	2.5 [1.6–3.9]	0.54	< 0.001		
Acute renal failure	2.7 [2.0–3.7]	0.60	< 0.001		
Blood transfusion	1.9 [1.3–2.8]	0.54	< 0.005		
Vasoactive drugs	2.2 [1.6–3.1]	0.59	< 0.001		
Mechanical ventilation	1.9 [1.3–2.5]	0.56	< 0.001		
ICU length of stay, days	1.03 [1.02–1.04]	0.62	< 0.001		
Sabadell score	5.1 [4.3–6.0]	0.86	< 0.001	3.8 [3.1–4.6]	< 0.001
High RDW	6.3 [3.9–9.9]	0.67	< 0.001	2.8 [1.7–4.6]	< 0.001

*SAPS3* Simplified Acute Physiologic Score 3, *RDW* red cell distribution width, *ICU* intensive care unit, *OR* odds ratio, *AUC* area under ROC

Sabadell score for prediction of ward mortality showed an AUROC of 0.863, and the inclusion of high RDW improved the AUROC to 0.890 (*p* < 0.05).

The best multivariable logistic regression model, which included age, SAPS3, and Sabadell score, yielded an AUROC of 0.901, with good calibration (Lemeshow goodness-of-fit = 0.118). Moreover, the addition of RDW slightly improved the accuracy of the model only marginally (AUROC 0.908; *p* = 0.047).

**Fig. 2:**
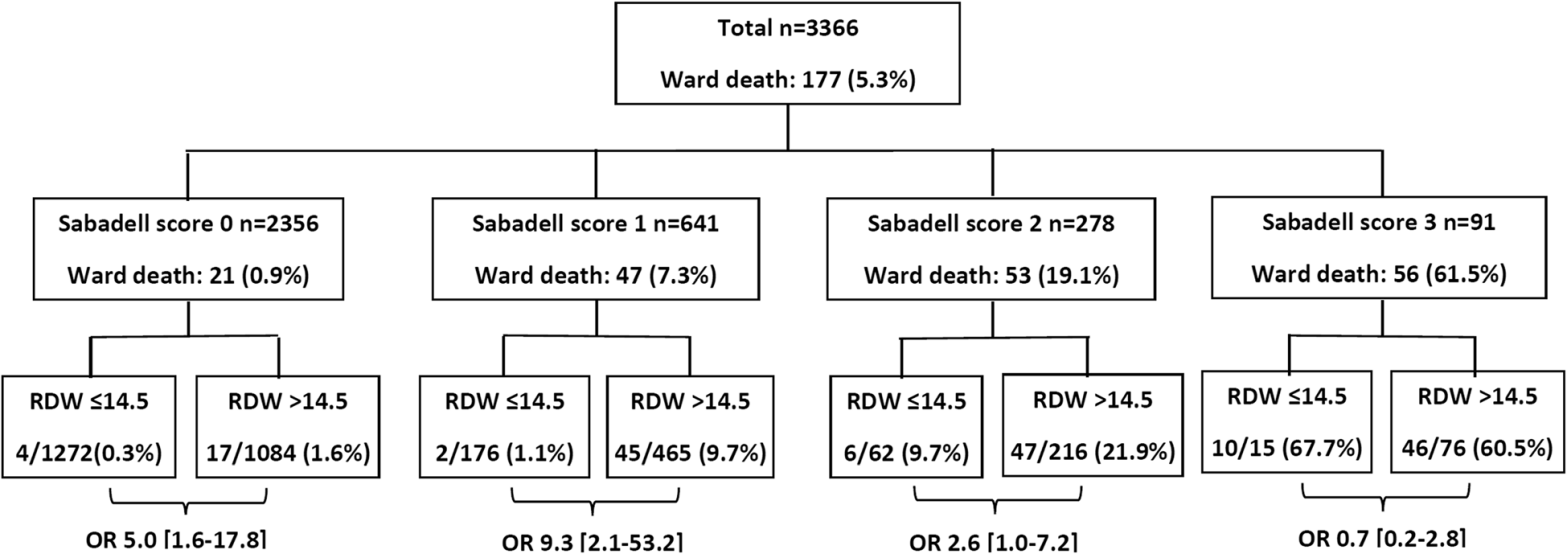
Ward mortality of patients at each Sabadell score group and associated red blood distribution width at ICU discharge

Additionally, when patients were grouped according to Sabadell score, high RDW identified patients with significantly higher ward mortality (Fig. 2). High RDW was significantly associated with higher ward mortality in patients with Sabadell score 0 (1.6% vs. 0.3%, OR 5.0 [1.6–17.8]), Sabadell score 1 (9.7% vs. 1.1%, OR 9.3 [2.1–53.2]), and Sabadell score 2 (21.9% vs. 9.7%, OR 2.6 [1.0–7.2]), but not in Sabadell score 3.

## Discussion

To our knowledge, this is the first study to show that high RDW is a marker of severity in patients discharged from the ICU to the wards. Additionally, high RDW identified patients with higher ward mortality at each level of prognosis depicted by the Sabadell score, except the most extreme SS3.

Our results in a wide sample of critically ill patients are in line with previous reports demonstrating high RDW as a marker of severity. Sadaka et al. [7] retrospectively studied 279 patients with septic shock and demonstrated that each quintile of RDW increase was associated with higher ICU mortality. Meynaar et al. [8] retrospectively studied 2915 patients in a mixed ICU and found that high RDW on admission was associated with hospital mortality; in multivariate analysis, RDW remained an independent risk factor for mortality after correction for APACHE II score, age, admission type, and mechanical ventilation (odds ratio 1.04 for each femtoliter of RDW). Bazick et al. [9] retrospectively studied a wide population of ICU patients and found that each quintile of RDW increase was associated with increased mortality and with an increased risk of acquiring a bloodstream infection. Zhang et al. [11] retrospectively studied 1539 ICU patients and found that high RDW on admission was associated with mortality, but its predictive performance was suboptimal (AUROC 0.62). Last year, Otero et al. [19] studied 500 critically ill surgical patients; 47% had high RDW on admission, and high RDW was associated with a twofold increase in mortality. In our study, ward mortality increased with RDW at ICU discharge. Beyond what would be expected considering the association between high RDW and severity of illness as defined by the SAPS3 score, the standardized mortality ratio was worse at higher RDW levels (Table 2).

The dynamic nature of RDW precludes firmly establishing the best time for RDW determination. Kim et al. [10] prospectively studied 329 septic patients and found that an increase in RDW > 0.2% from baseline during the first 72 h after hospitalization was significantly associated with higher mortality, even after adjusting for SOFA score and other covariates. Nevertheless, other investigators found no differences attributable to the evolution of RDW during the ICU stay [11]. We analyzed RDW at ICU discharge rather than at ICU admission, with the aim of attaining a better picture of the at-risk population just before discharge to the ward. To our knowledge, no other studies have focused on RDW at ICU discharge, although Purtle et al. [12] concluded that high RDW at hospital discharge is a robust predictor of subsequent all-cause patient mortality in critical care patients that survive hospitalization.

Another issue is whether high RDW can improve current mortality prediction models. In specific subgroups, some investigators [7, 20] found that adding high RDW resulted in statistically significant improvements in the predictive performance of severity scores, but that these increases were of little practical usefulness. In septic patients, Sadaka et al. [7] found that adding high RDW increased the AUROC from 0.69 to 0.77 in APACHE II. In patients treated with continuous renal replacement, Oh et al. [21] found that adding high RDW to SOFA increased the AUROC from 0.69 to 0.75. However, other investigators found that adding high RDW brought about only marginal improvement in APACHE II [8] and APACHE III [13]. In our study, adding high RDW improved the predictive performance of SAPS3 risk of death only marginally (AUC 0.778 vs. 0.798, data not shown).

Ward survival after ICU discharge remains an unresolved issue. In some scenarios, it appears to be a quality indicator, with higher mortality suggesting suboptimal treatment in the ward or premature ICU discharge. Nevertheless, some patients who survive the ICU stay have severe derangements that preclude significant recovery, and ward physicians and outreach teams would benefit from better classification of patients at ICU discharge [17, 18, 22]. A decade ago, Fernandez et al. [14, 15] devised a subjective score that classifies patients at ICU discharge into four groups with very different mortality in the ward, the Sabadell score. In the present study, adding high RDW to the Sabadell score improved the prediction of outcome in patients discharged to the ward. In each Sabadell score group except the worst (patients with null expected survival in the ward), patients with high RDW had substantially higher mortality than those with normal RDW.

Whether or not outreach teams should target patients with high RDW will depend on further investigations about the feasibility of treating the underlying conditions responsible for high RDW (inflammation, underperfusion states, or occult cancer).

### Limitations of the study

Our analysis of outcome at hospital discharge did not include patients transferred from the ward to other community hospitals or nursing homes. Nevertheless, in our public system, these transfers account for a minority of patients.

The lack of impact of high RDW in the more extreme SS3 patients may be related either to a small sample size or to the fact that mortality in this specific condition in mostly related to other variables (severe brain damage, refractory heart failure, and decisions to withhold/withdraw life support treatments).

We conclude that high RDW is a marker of severity in patients discharged from the ICU and improves the accuracy of the Sabadell score in predicting ward mortality except in the more extreme SS3. Whether outreach teams should target high patients with RDW remains to be determined.

## Abbreviations

APACHE: Acute physiologic and chronic health evaluation
AUROC: Area under the ROC curve
ICU: Intensive care unit
LOS: Length of stay
OR: Odds ratio
RDW: Red blood cell distribution width
ROC: Receiver-operator characteristic
SAPS3: Severity Acute Physiologic Score
SOFA: Sequential organ failure assessment
SS: Sabadell score
